# Elucidating Rheological Properties of Cementitious Materials Containing Fly Ash and Nanosilica by Machine Learning

**DOI:** 10.3390/nano14211700

**Published:** 2024-10-24

**Authors:** Ankang Tian, Yue Gu, Zhenhua Wei, Jianxiong Miao, Xiaoyan Liu, Linhua Jiang

**Affiliations:** 1 College of Civil and Transportation Engineering, Hohai University, Nanjing 211100, China; 15850605671@163.com (A.T.); liuxiaoyan@hhu.edu.cn (X.L.); hhulhjiang@163.com (L.J.); 2 Department of Ocean Science and Engineering, Southern University of Science and Technology, Shenzhen 518055, China; weizh@sustech.edu.cn; 3 Department of Civil and Environment Engineering, National University of Singapore, Singapore 117576, Singapore

**Keywords:** concrete, machine learning, rheology, solid waste, nanomaterials, nanosilica

## Abstract

Researching the rheology contributes to enhancing the physical and mechanical properties of concrete and promoting material sustainability. Despite the challenges posed by numerous factors influencing viscosity, leveraging machine learning in the era of big data emerges as a viable solution for predicting the general properties of construction materials. This study aims to create models to forecast the rheological properties of cementitious materials containing fly ash and nanosilica. Four models—Random Forest, XGBoost, ANN, and RNN (Stacked LSTM)—are employed to predict and assess shear rate versus shear stress and shear rate versus apparent viscosity curves. Through hyperparameter adjustments, RNN (Stacked LSTM) exhibits excellent performance, achieving a coefficient of determination (R^2^) of 0.9582 and 0.9257 for the two curves, demonstrating superior statistical parameters and fitting effects. The RNN (Stacked LSTM) exhibited a better generalization ability, suggesting it will be more reliable for future prediction in cementitious material viscosity.

## 1. Introduction

The cement industry [1] is responsible for nearly 8% of global carbon emissions due to high energy consumption [2] and the decomposition of limestone during clinker production. To reduce the environmental impact of cement production, the construction sector is increasingly utilizing industrial byproducts and waste as mineral admixtures to replace cement clinker and reduce its quantity in concrete formulations [3], thereby seeking a sustainable, low-carbon, and even durable future for cementitious materials [4]. For example, fly ash, which is a powdery byproduct collected from coal-firing power plants, is often used to partially replace cement to not only create economic and environmental benefits, but also improve the performance of concrete composites in terms of both early- and late-age properties (e.g., workability, pumpability and durability) [5]. In recent years, fly-ash-dosed concrete has further been admixed with nanosilica (NS) to significantly improve concrete performance by increasing its packing density and maximizing the pozzolanic effect [6]. The inclusion of fine particles such as nanosilica in concrete mixtures raises performance improvements [7], such as particle aggregation in cementitious materials, which are often addressed by the use of various of chemical admixtures to help improve particle dispersion [8].

While most of the published literature has been focusing on the strength development and durability of these so-called high-performance concrete (HPC) mixtures, very few studies have dealt with the rheological properties of mixtures containing fly ash and nanosilica [9]. Moreover, traditional methods for elucidating the rheological properties of cementitious materials are usually based on empirical models. Since the composition of HPC mixtures is far more complex than conventional concrete, the influence of the type and amount of various mineral and chemical admixtures on the rheological properties of HPC mixtures will be obtained via many tedious trial-and-error experiments using traditional methods. The lack of comparative analysis of the same experiments will make the already complex cement systems even less convincing in empirical modeling. Machine learning, as a very popular data analysis method, can be trained by the data under the same experimental conditions to obtain a reliable viscosity influence law of cementitious materials [10].

### 1.1. Rheology of Freshly Mixed Cement Paste

Rheology explores stress changes over time under external forces in objects [11]. Concrete processes, from mixing to hardening, involve rheological phenomena [12,13]. The rheological properties of concrete and the workability of fresh slurry significantly influence its overall performance and hardening, as the slurry is a fundamental constituent of concrete [14]. Mandal et al. [15] studied the rheological properties of self-compacting recycled aggregate concrete. Yin et al. [16] used waste glass as a cementitious material to study the rheological properties of high-performance concrete.

Most of the workability properties of traditional concrete are obtained through slump tests, but with the addition of fly ash and nanosilica [17]; to obtain a complete grasp of the rheological properties of freshly mixed concrete, it is necessary to start from the rheological mechanism to reveal the interactions between the various components of the concrete as well as the workability mechanism of the concrete [18]. From there, the relationship curve or relationship equation between the rheological properties of the concrete mix and the workability parameters in practical engineering applications can be established, and even a numerical simulation of concrete can be carried out. In rheology, the new version of concrete is a complex system [19]. In a study, the scientific rheological parameters provided by the rheometer were combined with the traditional engineering experience to establish a correlation, which can not only ease the difficulty of the concrete proportion design, but also optimize the performance of concrete [20,21]. Rheometers are devices that test the relationship between the torque and rotational speed of an object under shear, where continuous rotation is used to apply strain or stress to obtain a constant shear rate, and the torque due to flow deformation is measured when the shear flow tends to stabilize. Rheometers can characterize the rheological behavior of cementitious materials by two or more parameters compared to traditional collapse tests.

Predictive models can be implemented when experimental methods cannot be applied to real-world projects. It can be determined that the viscosity of cement slurry is mainly related by particle morphology according to the K-D viscosity formula [22]. The more angular particles decrease stacking density and impact the rheological properties [23]. Therefore, different mixing ratios, as well as the amount of mineral admixtures, are important factors affecting the rheological properties of fresh cement pastes. The accuracy of these models can be evaluated by comparing the predicted results with real data from experimental tests [24,25]. However, existing models struggle with modern multi-component cement mixes, leading to potential prediction failures due to increased complexity.

### 1.2. Machine Learning

In recent years, with the explosion of artificial intelligence, it has been used in many fields, and in the field of construction materials, machine learning, a branch of artificial intelligence, has been a great impetus to the research on the properties of cementitious materials [26,27]. Due to the variable mixing ratios of cementitious materials, many of their physicochemical properties are affected. The algorithmic models in machine learning can learn from the existing data to understand the implicit patterns of the complex properties of the material itself, and then predict the material properties for the unknown novel fit ratios [28,29,30]. Machine learning can be categorized into supervised and unsupervised learning based on the presence or absence of labels [31]. Supervised learning is where the model first learns the existing training dataset, and after learning from the training dataset, it then predicts from the test set and the output is given. Whereas unsupervised learning does not provide output data, the model is required to learn from it on its own. In the field of civil engineering, we mostly use supervised learning methods; currently, there are existing machine learning models which are widely used by researchers to predict the mechanical properties of concrete, such as strength or durability research, and we will usually use a variation of a ratio of the cementitious materials as the input parameter, such as the water–cement ratio, the amount of admixtures, and the type of mixing. Bello et al. [32] used machine learning to predict the water–cement ratio of concrete. Qian et al. [33] analyzed cement particles in cement paste based on machine learning. Beyond that, Rajendran et al. [34] diagnosed the compressive strength of cementitious materials through machine learning.

The machine learning algorithms used for general performance prediction of cementitious materials are broadly decision trees (DTs), Support Vector Machines (SVM), Random Forest (RF), artificial neural networks (ANNs), and so on. Nowadays, there are several review articles about machine learning algorithms that predict the properties of cementitious materials [35]. The role of machine learning in the field of construction materials is mainly to intelligently process the data while predicting the performance, which is mainly derived from probabilistic sources such as the coefficient of determination R^2^, the mean square error MSE, and the mean absolute error MAE [36]. These parameters can reflect the accuracy of the models we use in terms of visualized real data, and the predictions can be evaluated and compared between different models [37,38]. In addition, different input parameters have different effects on the output parameters, and we can use these mathematical metrics to analyze the importance of each input parameter to determine the weights of different input parameters.

Algorithmic models based on machine learning have also been preliminarily applied to study the rheological properties of cementitious materials; however, there are still some problems to be solved to further explore the rheological behavior of cementitious materials using ML, detailed as follows:

- Machine learning models require a large amount of data to learn from, which can improve the accuracy of predictions, but the rheological experiments have less data available and require the use of self-testing datasets, so there are fewer data sources available;
- In general, traditional rheological modeling is used to change a single variable to study the pattern of change, while machine learning can be used to verify the accuracy of prediction through the change in multiple sources of data, with some differences.

### 1.3. Research Significance

In this study, four machine learning models, namely extreme gradient boosting (XGBoost), Random Forest, artificial neural networks (ANNs), and recurrent neural networks (RNNs), were used to predict the relationship between the rheological properties of cementitious materials and their proportion design. The hyperparameters of the four models were adjusted to reach the optimal prediction level, and the prediction accuracy of the four algorithmic models was compared by a comprehensive assessment of the two sets of data, namely, the shear rate versus shear stress curve and the shear rate versus apparent viscosity curve, in the training dataset. Finally, the validity and reliability of the four models were verified by k-fold cross-validation.

## 2. Materials and Methods

### 2.1. Data Preparation

The data in this database are the curve of shear stress and apparent viscosity versus shear rate measured by the viscometer, and we select seven parameters as the input parameters, which are cement variety, water–binder ratio, fly-ash admixture, NS variety, NS admixture, total amount, and shear rate, and the output parameters are shear stress and apparent viscosity; a total of 168 sets of experimental data were measured, and the key hyperparameters of the model were adjusted to make the model achieve the best prediction through the training of this database. The new data were selected for the test for evaluating the model’s generalization to make sure that the test set was outside the training set. The interpretable limit characteristics of each parameter are shown in Table 1 [39].

### 2.2. Experimental Test Methods

**Figure 1:**
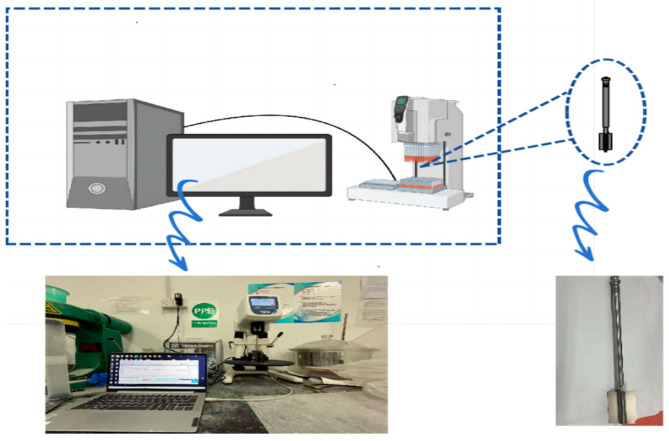
The RST-SST soft–solid-type rheometer, and the VT-50-25 rotor.

The experimental apparatus used for this rheological experiment was an RST-SST soft–solid-type rheometer, as shown in Figure 1. The rotor model used was VT-50-25, and this experiment was conducted at 20 ± 0.5 °C.

**Figure 2:**
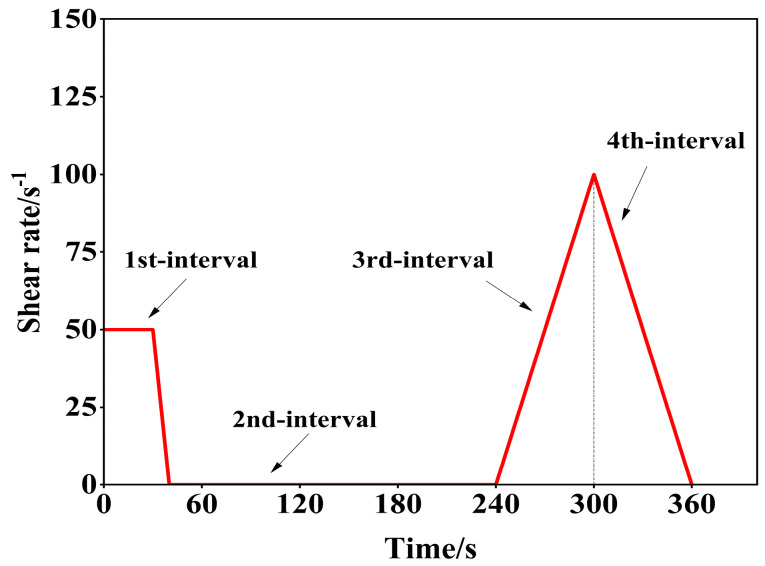
Measurement program diagram.

The following are the test steps:

1. Firstly, mix the micronized powder with cement according to the proportion of cementitious materials.
2. Prepare cement slurry with a clean slurry machine, wet the mixing pot in advance, pour dry materials with different ratios into the mixing pot, stir slowly for 60 s, stir quickly for 60 s, pause for 60 s, scrape the slurry on the wall during this period, then stir quickly for 20 s, for a total of 200 s.
3. After mixing, quickly take 100 mL of freshly mixed cement paste and put it in the measuring cup, and test its rheological properties with a rheometer.
4. Pre-shear at a constant rate of 50 s^−1^ for 30 s, then decrease the rate from 50 s^−1^ to 0 s^−1^ in 10 s, and then let it stand for 200 s, then increase the shear rate from 0 s^−1^ to 100 s^−1^ in 60 s, and then decrease the shear rate from 100 s^−1^ to 0 s^−1^ in 60 s. The diagram of the measurement procedure is shown in Figure 2. Similar procedural diagrams of this kind are extensively employed in rheological model studies [40].

### 2.3. ML Models

#### 2.3.1. Random Forest

The Random Forest model is used in regression or classification problems and the field of construction materials. It is mostly used for regression prediction of the general properties of cementitious materials. In the regression problem, the Random Forest algorithm builds multiple decision trees, each of which is constructed by randomly selecting the samples and features. The decision tree recursively divides the dataset through the decision nodes until it obtains the pure leaf nodes. The leaf nodes are the predicted values we need, there are two random processes in Random Forest, namely bootstrap and random selection of features; bootstrap ensures that each tree selects different data, and random selection of features reduces the correlation between trees. When aggregating the prediction results, we take the average value to solve the regression problem. Suenaga et al. [41] used Random Forest to predict the anchor shear resistance. The number of decision trees (n estimators) used in advance for this model is 100 and the maximum depth of the decision tree (max depth) is 10.

#### 2.3.2. XGBoost

The XGBoost algorithm is a multi-classifier based on the idea of boosting and the GBDT algorithm, which integrates multiple weak classifiers, performs integrated learning, and uses multiple decision trees for prediction; each time a new decision tree is used to fit the residuals of the previous decision tree, and at the same time as the new decision tree produces new residuals, a new decision tree is constructed to fit the residuals, and so on and so forth. Until the predefined conditions are reached, these weak classifiers are combined into a total classifier by weighted summation, which is the principle of the XGBoost algorithm. Safhi et al. [42] predicted the self-consolidating concrete properties by using the XGBoost algorithm. Wang et al. [43] predicted the corrosion problem of microbial concrete in sewers by using XGBoost. The number of decision trees (n estimators) used in advance for this model is 100, the maximum depth (max depth) of the decision tree is 5, and the learning rate (learning rate) is 0.1.

#### 2.3.3. Artificial Neural Network

**Figure 3:**
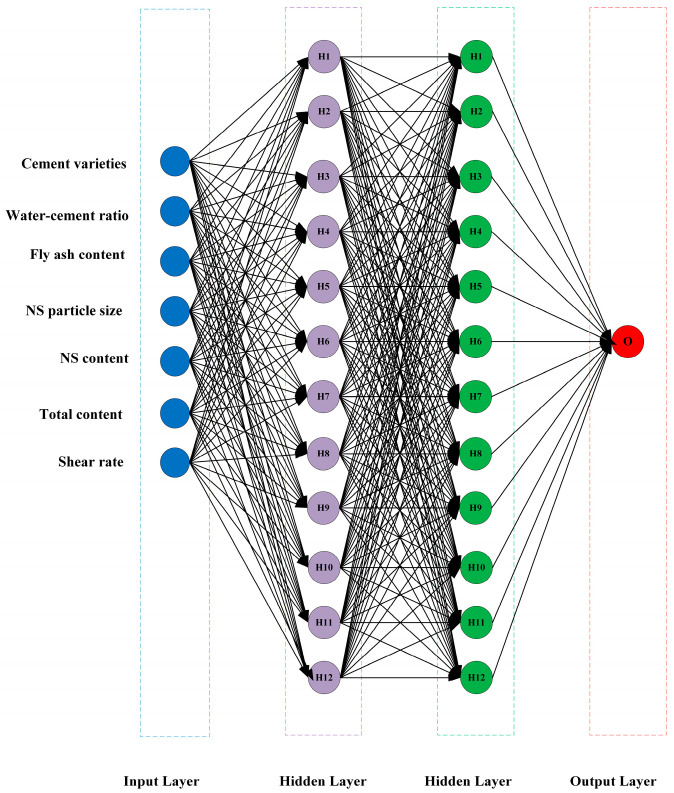
A schematic diagram of an ANN.

Artificial neural networks, as an important algorithm for data mining, have been used by many to predict the general properties of cementitious materials such as strength prediction as well as durability prediction [44]. Artificial neural networks mimic the structure of the biological human brain to analyze the data. They are divided into the input layer, the hidden layer, and the output layer. Each layer has a certain number of neurons and each neuron has an activation function. The common activation functions are the sigmoid function, ReLU function, tanh function, etc. Each neuron is weighted and summed according to the inputs and weights and processed by the activation function to produce the output. Mohammed et al. [45] evaluated and analyzed the compressive strength of fly-ash-modified cement mortar based on an ANN, Nasir Uddin et al. [46] used an ANN to analyze the compressive strength of engineered cementitious composites (ECCs) for the prediction of the PVA fiber effect in ECCs. Since few artificial neural network models have been used to predict the rheological properties of cementitious materials, the predictive ability of artificial neural networks in this system is not clear. The artificial neural network model developed in this study (Figure 3) contains 2 hidden layers, each containing 12 hidden nodes.

#### 2.3.4. Recurrent Neural Network

A recurrent neural network (RNN) is a kind of neural network model for processing temporal sequence data, which has shown strong potential and wide application in processing temporal sequence data through its unique recurrent structure. In rheological experiments, the shear rate varies with time. So, as a time-varying parameter of cementitious materials, the RNN model may be more effective in predicting the rheological properties of cementitious materials. Jiang et al. [47] used an RNN to predict the adiabatic temperature rise of complex concrete, and Liu et al. [48] used an RNN to predict the energy consumption of cement systems.

**Figure 4:**
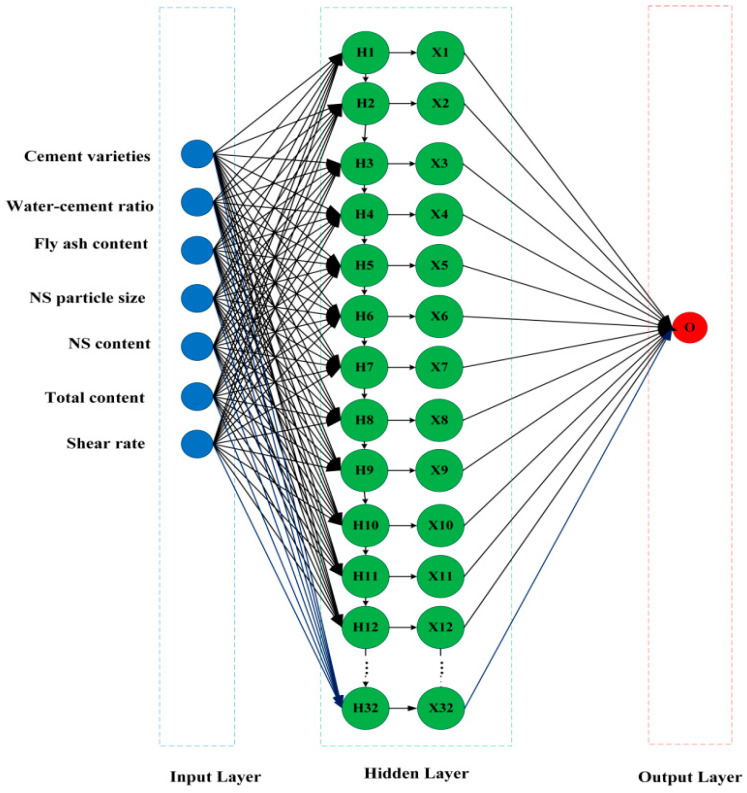
A schematic diagram of a simple RNN.

**Figure 5:**
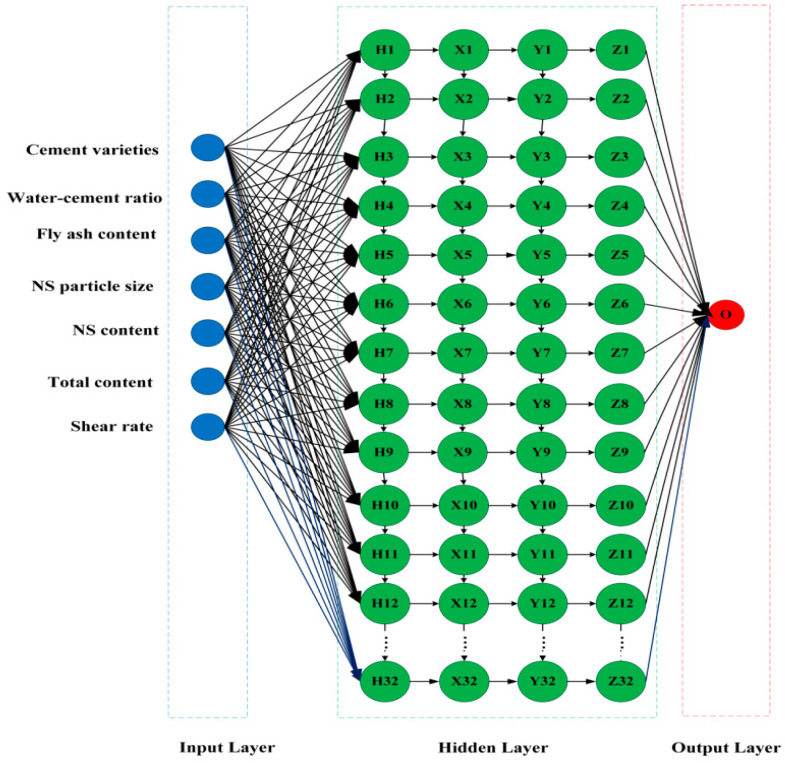
Schematic diagram of Stacked LSTM.

**Figure 6:**
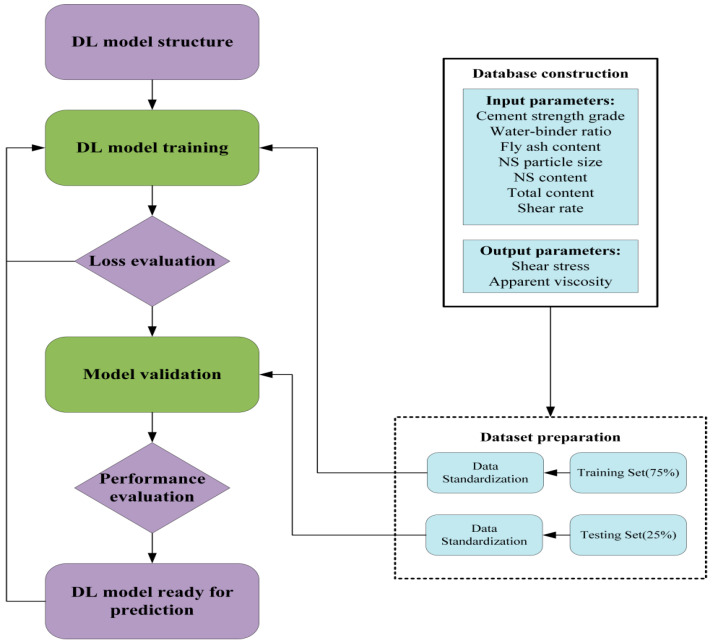
Methodology of ML model development.

For simple RNN model, it is easy to cause the gradient disappearance, which means that the model can only have a short-term memory, and from this we use LSTM layer; unlike the simple RNN model, LSTM uses a transmission band, which can easily transfer past information to the next moment, so that there is a longer memory, which is very important for time-series data. In terms of prediction accuracy, LSTM will also perform better than simple RNN. Simple RNN is shown in Figure 4. In this study, a prediction model for the rheological properties of freshly mixed cement paste based on Stacked LSTM is developed, as shown in Figure 5. The rheological properties of freshly mixed cement paste under different mix proportions are predicted through the main steps illustrated in Figure 6.

## 3. Results and Discussion

### 3.1. Predictive Performance Indicators

In order to express the prediction accuracy of the model, we generally use some statistical indicators, such as coefficient of determination (R^2^), mean absolute error (MAE), root mean squared error (RMSE), and mean absolute percentage error (MAPE). RMSE is the difference between the predicted value and the true value, then the sum of squares, then the average, and finally the square root, while MAE is the difference between the predicted value and the true value, then the absolute value, and then the average; both MAE and RMSE are the indicators that show the difference between the predicted value and the true value, and the smaller the MAE and RMSE are, the more accurate the prediction of the model will be. The coefficient of determination R^2^ can be understood as using the mean value as the error benchmark to see the relationship between the prediction error and the mean benchmark error. The closer R^2^ is to 1, the better the independent variable explains the dependent variable in the regression analysis. MAPE is a relative error metric that uses absolute values to avoid the offset between positive and negative errors; the lower the value of MAPE, the lower the prediction discrepancy. The MAPE can be used to compare the forecasting accuracy of time-series data models. The following are the formulas for several statistical metrics:

(1) $$RMSE=\sqrt{\frac{\sum_{i=1}^{n}{\left(y_{i}^{\prime }-y_{i}\right)}^{2}}{n}}$$

(2) $$MAE=\frac{1}{n}\sum_{i=1}^{n}\left|y_{i}^{\prime }-y_{i}\right|$$

(3) $$R^{2}=1-\frac{\sum_{i=1}^{n}{\left(y_{i}^{\prime }-y_{i}\right)}^{2}}{\sum_{i=1}^{n}{\left(y_{i}-\bar{y}\right)}^{2}}$$

(4) $$MAPE=\frac{1}{n}\sum_{i=1}^{n}\left|\frac{y_{i}^{\prime }}{y_{i}}\right|\times 100$$

where $y_{i}^{\prime }$, denotes the predicted value of the output data, $y_{i}$ denotes the true value of the output data, $\bar{y}$ is the average of the true values of the output data, and $\bar{y^{\prime }}$ denotes the average of the predicted values of the output data.

### 3.2. Evaluation of the Predictive Performance of Four Models for Shear Rate–Shear Stress Curves

The shear rate and shear stress curves have a great influence on the rheological properties of cementitious materials [49], mainly in the viscoelastic and rheological properties of the material [50]. The change in shear rate affects the deformation behavior of the material, and the curve can be demonstrated by the rheological curve; by changing the applied shear rate, different shear stress values are obtained, and these data can be used to construct the yield stress curve, which shows the rheological properties of the material, which is of great significance for the design and engineering applications of the material.

Firstly, for XGBoost, the prediction accuracy of R^2^ is 0.8203, MAPE is 15.61, RMSE is 4.5244, and MAE is 4.4226, and for the Random Forest model, after adjusting the general hyper-parameter settings, the prediction accuracy of R^2^ is 0.8327, MAPE is 15.39, RMSE is 4.3301, and MAE is 3.4311. For the prediction of the shear rate and shear stress curves, it can be seen from the above four indexes that both models have an average prediction accuracy, and as in the integrated learning methods, the prediction accuracy of both models is average. For the prediction of the shear rate and shear stress curves, the prediction accuracies of the two models are general, as shown by the above four indexes. Random Forest and XGBoost use multiple models combined with a decision tree as the base model to improve the prediction performance. Due to the fact that the shear rate–shear stress curve is mostly a nonlinear problem, it is easy to see that the prediction accuracy of the integrated learning method is not high. So we then use two neural network models for prediction.

**Figure 7:**
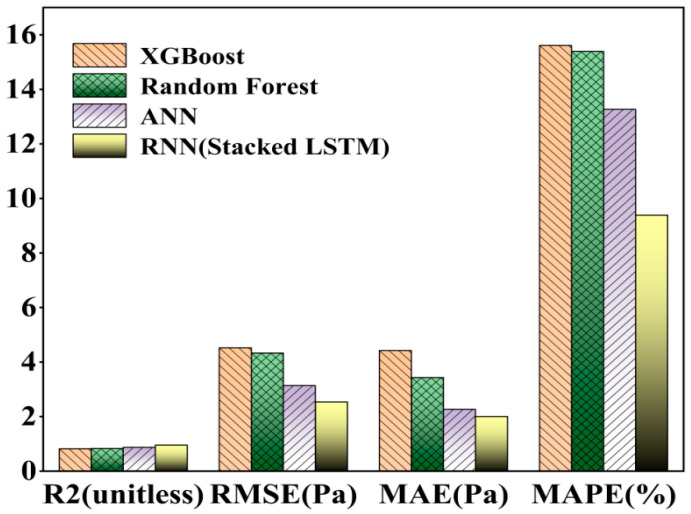
A comparison of the shear rate–shear stress curves’ statistical parameters.

Neural network models simulate complex multi-dimensional functions through the combination of multi-layer neurons and activation functions. The visualization of neural networks is generally expressed by the loss value and the number of iterations; epoch refers to the number of times the model has been trained on a training set, and in general, the parameters of the model will be updated once for each iteration until the model converges or reaches a particular condition. At the same time, with the increase in epoch, the loss value will be reduced accordingly. The epoch and learning rate are complementary to each other, if the epoch is set to a large case, and the learning rate is very small, it will make the model learn very carefully step by step. Although this sounds very good, it is easy to cause overfitting, so the actual situation needs to be considered in the parameter settings. In the ANN model, the prediction accuracy is 0.8729, the MAPE is 13.27, the RMSE is 3.1385, and the MAE is 2.2688. For the RNN (Stacked LSTM), the prediction accuracy is 0.9582, the MAPE is 9.39, the RMSE is 2.5377, and the MAE is 2.0057. Even under a lower iteration number, the prediction performance is still better. The prediction performance is still good, which reflects the high accuracy of the RNN model for time-series data prediction. The model predictions are shown in Table 2, and the parameters are visualized in Figure 7.

**Figure 8:**
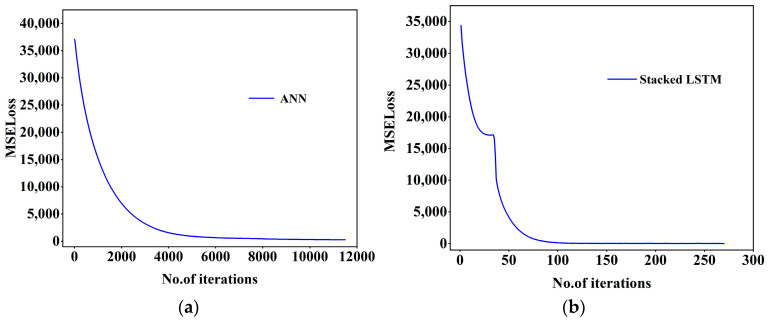
Losses in the training state of the model: (**a**) ANN; (**b**) Stacked LSTM.

**Figure 9:**
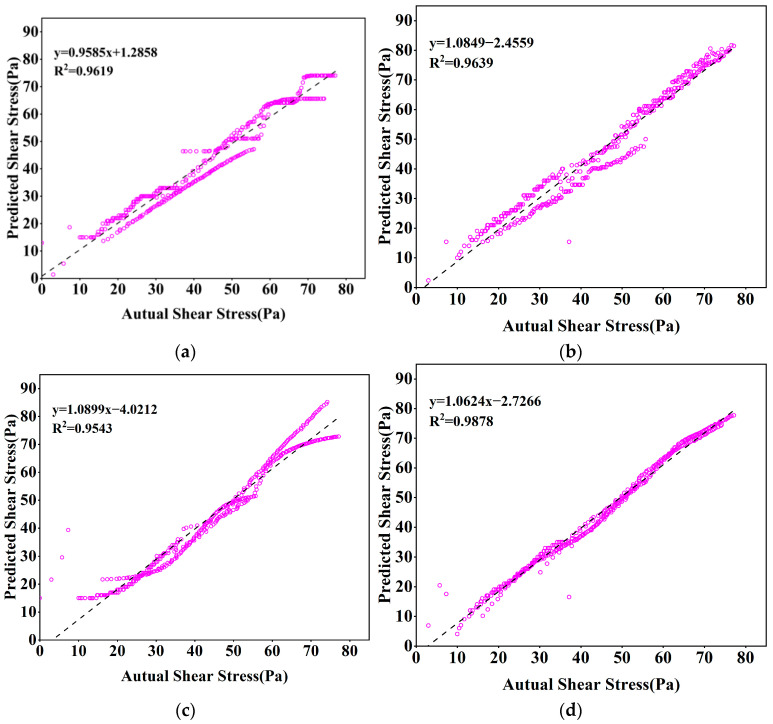
Correlation between actual and predicted shear stresses: (**a**) Random Forest; (**b**) XGBoost; (**c**) ANN; (**d**) RNN (Stacked LSTM).

**Figure 10:**
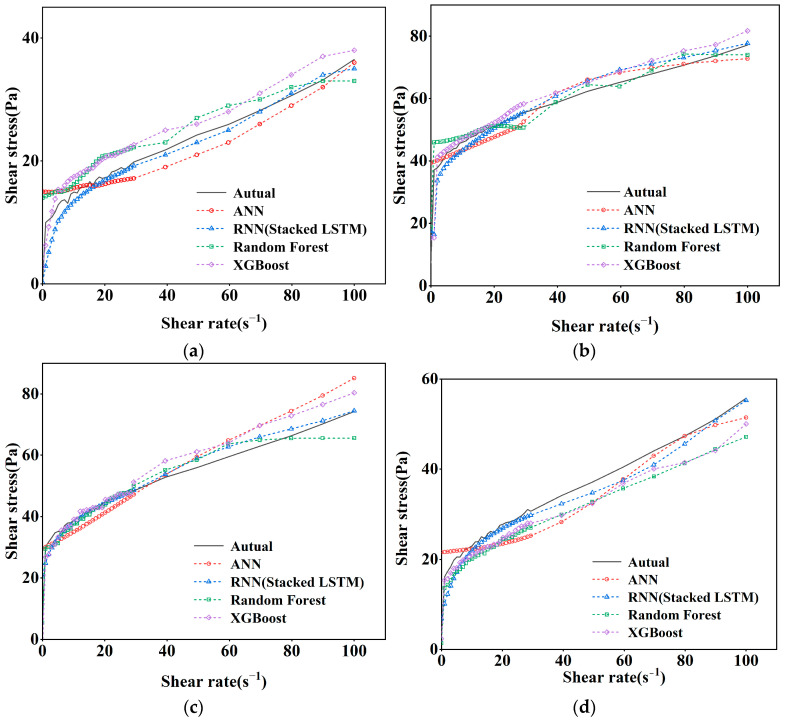
Shear rate–shear stress prediction curves for different mixing ratios: (**a**) P.O52.5 W/C0.55 FA0% nano-SiO_2_ 0%; (**b**) P.O52.5 W/C0.55 F10% 30 nm nano-SiO_2_ 2%; (**c**) P.O42.5 W/C0.45 F15% 30 nm nano-SiO_2_ 1%; (**d**) P.O52.5 W/C0.55 F10% 50 nm nano-SiO_2_ 0.5%.

From Figure 8, it can be concluded that in the process of model training, the final loss values are similar, but the number of epochs required by RNN (Stacked LSTM) is less, so the training time of the model will also be reduced, which also indicates that the RNN (Stacked LSTM) model is more advantageous. From Figure 9, it can also be seen that the actual shear stress is different from that predicted by the model, but the RNN (Stacked LSTM) model can obtain better fitting results by understanding the relationship between the input parameters and the output parameters. The prediction performance of the four models can be visually compared in Figure 10. When the shear rate is in the range of 0–20, the prediction accuracies of the four models are close to the actual values, and the accuracies of the models vary in the range of 20–100, in which most of the data points predicted by the RNN (Stacked LSTM) model are close to the actual values of shear stress. The Stacked LSTM model can be considered a better model for predicting the shear rate versus shear stress curve of freshly mixed cement paste.

### 3.3. Evaluation of the Predictive Performance of Four Models for Shear Rate–Apparent Viscosity Curves

The shear rate versus apparent viscosity curve is a key parameter in describing the rheological properties of cementitious materials, and the curve reflects the trend in the cement paste during the shear process [51]. At a low shear rate, the particles and molecules have enough time to rearrange, which makes the material show excellent viscosity [52]; meanwhile, at a high shear rate, the time for the particles and molecules to rearrange is reduced, which leads to a decrease in the viscosity of the material [53]. The shear rate versus apparent viscosity curve is an important indicator of the rheological properties of materials, which plays a very important role in the regulation and optimization of engineering applications.

**Figure 11:**
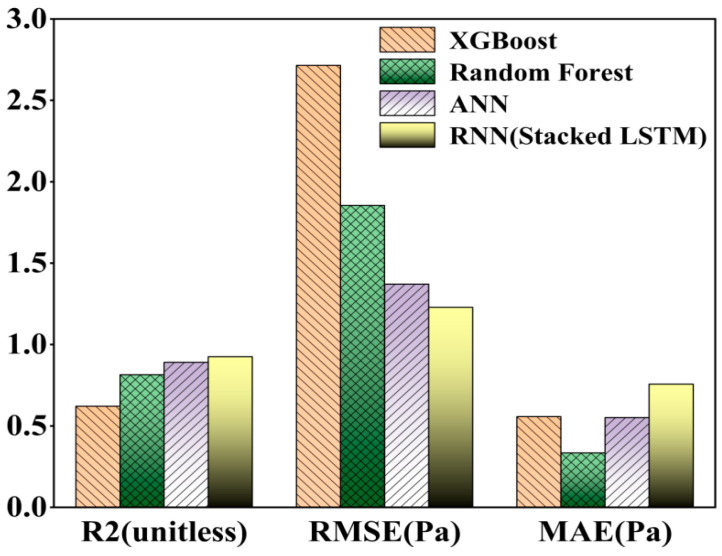
A comparison of the shear rate–apparent viscosity curves’ statistical parameters.

The same four models, without changing the input parameters, adjusted the output parameters to predict and evaluate the shear rate and apparent viscosity curves of the new version of the cement paste, according to the predictive statistical parameters in Table 3, it can be seen that the RNN (Stacked LSTM) model has the highest accuracy in predicting the apparent viscosity of the freshly mixed cement paste, and the coefficient of determination, R^2^, reaches 92%, which is mainly due to the fact that the LSTM model is more capable of memorizing the time-ordered data; at the same time, all of the overall performances of the deep learning neural network model are better than that of the integrated learning method of machine learning, and the visualization is shown in Figure 11.

**Figure 12:**
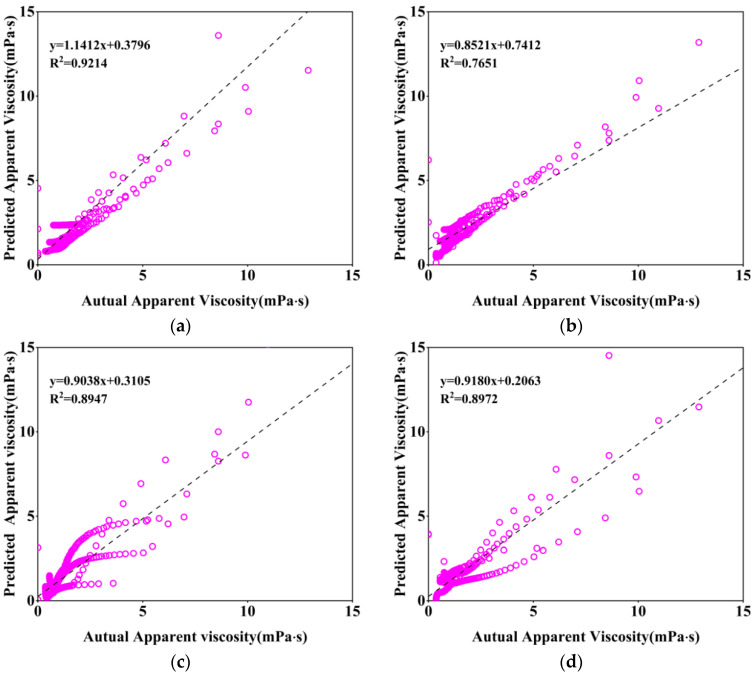
Correlation of actual apparent viscosity with predicted apparent viscosity: (**a**) Random Forest; (**b**) XGBoost; (**c**) ANN; (**d**) RNN (Stacked LSTM).

**Figure 13:**
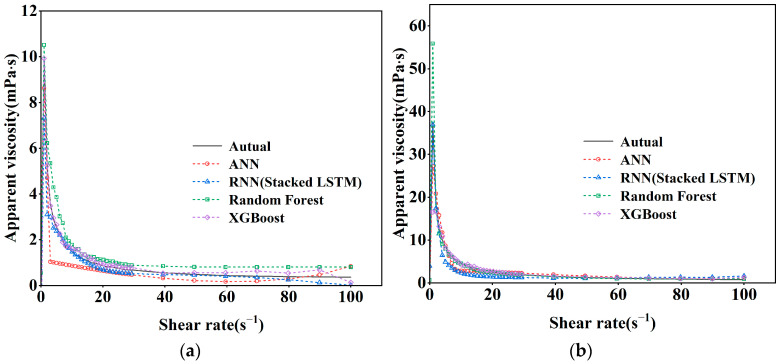

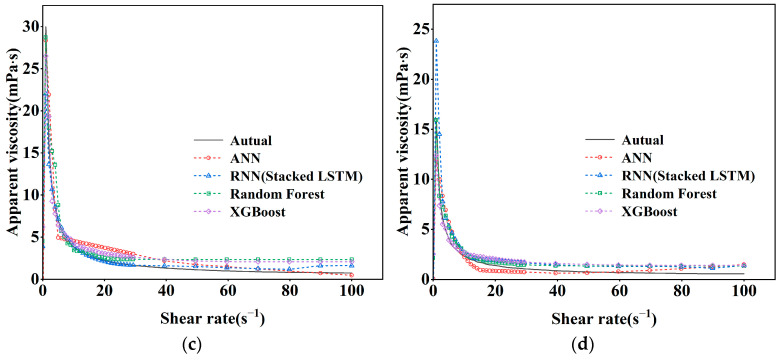
Shear rate-apparent viscosity prediction curves for different mixing ratios: (**a**) P.O52.5 W/C0.55 FA0% nano-SiO_2_ 0%; (**b**) P.O52.5 W/C0.55 F10% 30 nm nano-SiO_2_ 2%; (**c**) P.O42.5 W/C0.45 F15% 30 nm nano-SiO_2_ 1%; (**d**) P.O52.5 W/C0.55 F10% 50 nm nano-SiO_2_ 0.5%.

As can be seen in Figure 12, the apparent viscosity predicted by the RNN (Stacked LSTM) model is the most similar to the actual value. Figure 13 shows the comparison between the predicted and actual apparent viscosity curves of the four models with different mixing ratios, and the prediction accuracies of the four models are similar at the shear rate of 0–30 s^−1^, but the prediction differences between the four models appear at the stage of 30–100 s^−1^! However, at the stage of 30–100 s^−1^, the difference between the four models is obvious, and the predicted curves (blue) of the RNN (Stacked LSTM) model are the closest to the actual curves (black), so it can be seen that the RNN (Stacked LSTM) model can be regarded as a better model for the prediction of the shear rate vs. the viscosity curves of freshly blended cement pastes.

### 3.4. Limitations of the Study

In this study, the influence of various factors on rheological properties was simulated by changing the water–cement ratio, cement variety, fly-ash content, nano-SiO_2_ content, and nano-SiO_2_ particle size These factors are only considered through the physical effect on the rheological properties, but there is also a need to examine the chemical reaction and the hydration process on the rheological properties. In addition, this study is based on rheological experiments, and some microscopic experiments to analyze the mechanism can be included in the future.

## 4. Conclusions

The aim of this study is to construct a simple rheological performance model for fresh cement slurry, and to investigate the effects of the water–cement ratio, cement variety, fly-ash dosage, and SiO_2_ nanoparticles and dosage on the rheological performance of the slurry. The following four kinds of machine learning models were constructed: Random Forest, XGBoost, ANN, RNN (Stacked LSTM); the shear rate and shear stress curves and the shear rate and apparent viscosity curves obtained from the rheological experiments were used as the evaluation indexes, two curves were predicted by the above four kinds of machine learning models with the given input data, and the performance of the models was improved by adjusting the hyper parameters. The following conclusions are drawn:

1. In predicting the shear rate versus shear stress curves, the RNN (Stacked LSTM) model (R^2^ = 0.9582, RMSE = 2.5377, MAE = 2.0057) exhibits superior predictive accuracy compared to Random Forest (R^2^ = 0.8327, RMSE = 4.3301, MAE = 4.3301), XGBoost (R^2^ = 0.8203, RMSE = 4.5244, MAE = 4.5244), and ANN (R^2^ = 0.8729, RMSE = 3.1385, MAE = 3.1385).
2. In predicting shear rate versus apparent viscosity curves, the RNN (Stacked LSTM) model (R^2^ = 0.9257, RMSE = 1.2288, MAE = 0.7571) outperforms the Random Forest (R^2^ = 0.8146, RMSE = 1.8547, MAE = 0.3340), XGBoost (R^2^ = 0.6210, RMSE = 2.7148, MAE = 0.5578), and ANN (R^2^ = 0.8912, RMSE = 1.3711, MAE = 0.5518) models in terms of predictive performance.

Combining the predictions of both curves, RNN (Stacked LSTM) is a better model to predict the rheological properties of freshly mixed cement paste, and for time-series data, RNN (Stacked LSTM) performs better. In future research, this database can be expanded by incorporating additives and additional input data to enhance the model’s interpretability for subsequent studies.

## Figures and Tables

**Table 1:** The limitation values of seven explainable features.

Ingredients or Features	The Range of Values	
	Minimum	Maximum
Cement strength grade (MPa)	42.5	52.5
Water–binder ratio (%)	0.35	0.55
Fly-ash content (g)	10	30
NS particle size (nm)	30	50
NS content (g)	1	4
Total content (g)	200	200
Shear rate (s^−1^)	0	100

**Table 2:** The statistical parameters for the prediction of shear rate vs. shear stress curves.

ML Model	R^2^ (Unitless)	RMSE (Pa)	MAE (Pa)	MAPE (%)
XGBoost	0.8203	4.5244	4.4226	15.61
Random Forest	0.8327	4.3301	3.4311	15.39
ANN	0.8729	3.1385	2.2688	13.27
RNN (Stacked LSTM)	0.9582	2.5377	2.0057	9.39

**Table 3:** The statistical parameters for the prediction of shear rate vs. apparent viscosity curves.

ML Model	R^2^ (Unitless)	RMSE (Pa)	MAE (Pa)
XGBoost	0.6210	2.7148	0.5578
Random Forest	0.8146	1.8547	0.3340
ANN	0.8912	1.3711	0.5518
RNN (Stacked LSTM)	0.9257	1.2288	0.7571

## Data Availability

Data will be made available on request.
